# Constitutive Overexpression of an NB-ARC Gene from Wild Chinese *Vitis quinquangularis* in *Arabidopsis thaliana* Enhances Resistance to Phytopathogenic Oomycete and Bacteria

**DOI:** 10.3390/ijms25063221

**Published:** 2024-03-12

**Authors:** Xiangjing Yin, Qian Zha, Pengpeng Sun, Xiaojun Xi, Aili Jiang

**Affiliations:** 1 Forest and Fruit Tree Research Institute, Shanghai Academy of Agricultural Sciences, Shanghai 201403, Chinazhaqian@saas.sh.cn (Q.Z.); sunpp97@163.com (P.S.);; 2 Shanghai Key Labs of Protected Horticultural Technology, Shanghai Academy of Agricultural Sciences, Shanghai 201403, China

**Keywords:** grape, *Plasmopara viticola*, disease resistance, SA signaling pathways

## Abstract

Resistance (R) genes were used to recognize pathogen effectors directly or indirectly in plants and activate defense signal pathways. Most of these R proteins consist of a nucleotide-binding adaptor (NB-ARC) domain, a leucine-rich repeat (LRR) domain and some also have a coiled-coil (CC) structure. In this study, we cloned a gene which encodes the CC-NB-ARC-LRR R protein (*VqCNL*) from Chinese wild grapevine *Vitis. quinquangularis* accession ‘Dan-2’. The transcript of *VqCNL* was obviously induced by inoculation with *Plasmopara viticola* and the salicylic acid (SA) treatment. The results of sequence analysis showed that the *VqCNL* gene contained a CC domain at the N-terminus, along with an NB-ARC and an LRR domain at the C-terminus. We transferred this gene into wildtype *Arabidopsis* and treated transgenic lines with *Hyaloperonospora arabidopsidis* (*Hpa*) and *Pseudomonas syringae* pv. *tomato* DC3000 (*Pst* DC3000); the results demonstrated that *VqCNL* promotes broad spectrum resistance to pathogens. Furthermore, qPCR analysis displayed that *VqCNL* may display a significant function in disease resistance via activating SA signaling pathways. In general, these conclusions primarily demonstrated that *VqCNL* enhances the disease resistance level in plants and contributes to future research of the *R* gene identification for grape breeding biotechnology.

## 1. Introduction

Exposed to multiple environmental stresses like extreme temperatures, drought, insects and pathogen infections in their lifetime, plants can generate various adaptive mechanisms to avoid or tolerate environmental changes during their entire life cycle [1,2]. In the process of response to biotic stresses, physical and chemical barriers are the first line of defense against pathogens. Furthermore, there are also inducible molecular defense strategies in plants which play important roles for plant growth and development. These defense barriers include a complex immune system called innate immunity that protects plants from pathogens [3]. In the presence of pathogen infection, cell membrane proteins of pattern recognition receptors (PRRs) recognize pathogen-associated molecular patterns (PAMPs) and activate a basic immune response called PAMP-triggered immunity (PTI) [4]. To suppress the PTI response, pathogens have evolved specialized secretory systems that transport effectors into plant cells. These toxic effectors are recognized by the polymorphic intracellular receptor complex named nucleoside binding/leucine-rich repetitive sequences (NB-LRR). In order to overcome these pathogen effectors, plants have developed resistance (R) proteins that directly or indirectly recognize pathogen effectors and activate the second defense line called effect-triggered immunity (ETI) [5,6,7]. ETI can trigger a series of robust defense reactions, including the production of pathogen-related proteins 1, reactive oxygen species (ROS) burst, plant antitoxins and hypersensitive response (HR). HR is a type of programmed cell death (PCD) that reduces the proliferation of attacking pathogens [8,9]. Playing a significant role in the process of plant disease resistance, R proteins can recognize pathogen effectors and activate disease resistance response. Therefore, revealing the mechanism of interaction between R protein and pathogen effector will help develop new methods for controlling and preventing pathogen invasion.

In 1992, the first *R* gene was cloned and published. After that, the number of functional R genes increased gradually. Identified from maize, *Hm1*, which encodes a disease resistance enzyme, controls race-specific resistance to the fungus *Cochliobolus carbonum* [10]. Following the cloning of *Hm1*, gene *Pto* and *Cf-9* were cloned from tomato, genes *N* from *Nicotiana tabacum*, and *RPS2* from *Arabidopsis thaliana* [11,12,13,14]. From then on, the cloning of hundreds of *R* genes has been published. A total of 314 *R* genes had been cloned and studied [15]. These disease resistance genes affect the resistance of related pathogens which can be located inside or outside plant cells, including bacteria, viruses, fungi, oomycetes, even nematodes and insects. Although there are great differences between these pathogens and their pathogenic molecules, most of the resistance proteins that play a role in recognition can be classified according to their conserved domains. NBS-LRR is a class of intracellular receptor proteins with nucleotide binding sites and leucine-rich repeats [16]. Multiple conserved subdomains of NB-ARC like Walker A, Walker B, P-loop, GLPL, MHD and RNBS-A–D were characterized in R proteins. Based on the structural analysis and molecular functions of the NB-ARC domain, it might display the function of a molecular switch to regulate multiple disease resistant signaling pathways throughout conformational changes [17,18,19,20,21].

The NBS domain was widely used for the identification and classification of plant resistance genes, and contains conserved motifs. According to phylogenetic studies of the structural difference at the N-terminal of the NBS domain, NBS-LRR proteins were mainly divided into two categories in plants: the TNL type and the non-TNL type [22]. TIR-NB-ARC-LRR or TNL proteins mean those that contain a Toll and human interleukin-1 receptor (TIR) domain. A common class of these proteins is that they contain a conversed coiled-coil (CC) structure at the N-terminal. The structure of the CC domain consists of multiple α-helices which form around other subdomains to form a super-coil. Like the TIR domain, the CC domain displays functions of recognizing connected effectors to trigger a series of downstream signaling responses [23,24]. The Arabidopsis RPM1 protein is a typical CC-NBS-LRR protein which is involved in indirect intracellular recognition of the *P. syringae* effectors (AvrRpm1) to activate resistance responses [25]. The CC domain of NRG1 from *Arabidopsis* belongs to CNL proteins which have been proved to independently induce a defense response [26]. CC-NB-LRR proteins in the plant may form cytoplasmic transduction–transcriptional regulatory processors that regulate the defense response by activating the expression of defense-related genes. The N-termini of the CC and TIR domains are supposed to mediate downstream immune responses and display key roles in the cell death signaling pathways [17].

Grapevine has been planted worldwide and recognized as one of the high economic value fruit crops. Nevertheless, the cultivated planted grape species is vulnerable to a variety of pathogens, threatening substantial reductions in production and quality. Downy mildew (DM), one of the most serious diseases to *V. vinifera*, caused by the oomycete called *Plasmopara viticola*, is documented to attack grapevine leaves, employing haustoria to infiltrate through the stomata [27]. Pinpointing the genes that encode NB-ARC-LRR proteins capable of conferring resistance to *P. viticola* is essential for deepening research into grapevine—*P. viticola* dynamics and for generating novel resistance methods to combat downy mildew. China is a major origin of grapes, and some of the wild species found in the country have been proved highly resistant to the pathogen. Therefore, Chinese wild grape varieties have the potential to provide a plentiful resource to further understand the molecular mechanisms of grape *P. viticola* interaction [28].

The Chinese wild grapevine species *Vitis quinquangularis* ‘Dan-2’ has been demonstrated to exhibit a relatively high level of resistance to multiple microorganisms, and particularly *P. viticola* [29]. Nevertheless, little research has been identified and functionally characterized on NB-ARC-LRR genes’ involvement in DM resistance in Chinese wild Vitis species. Thus, to enhance comprehension of the defense mechanisms against *P. viticola*, we previously employed various analytical methods to explore the resistance characteristics in Chinese wild grapevine upon inoculation with *P. viticola* [30]. Within the set of genes activated in response to the pathogen, one was identified as potentially encoding a CC-NBS-LRR domain protein. The current study extracted the full-length coding sequence of this gene, designated as *VqCNL*, and conducted a functional characterization via its constitutive heterologous expression in *Arabidopsis*.

## 2. Results

### 2.1. Cloning and Sequence Analysis of VqCNL

**Figure 1:**
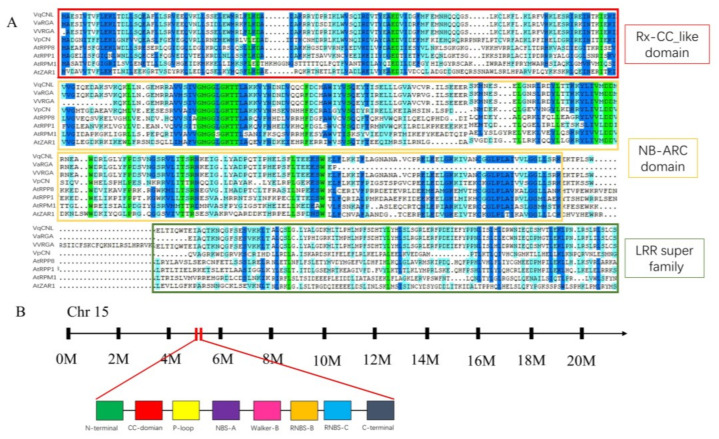
Sequence analysis of *VqCNL*. (**A**) Multiple sequence alignment of RX-CC like, NB-ARC and LRR domains from *VqCNL, AtRPP8*, *AtRPP13*, *AtRPM1*, *AtZAR1*, *VvRGA* and *VaRGA*. (**B**) Schematic map of the chromosomal location of *VqCNL*, as well as the significant conserved motifs and domains in the deduced *VqCNL* amino acid sequence.

**Figure 2:**
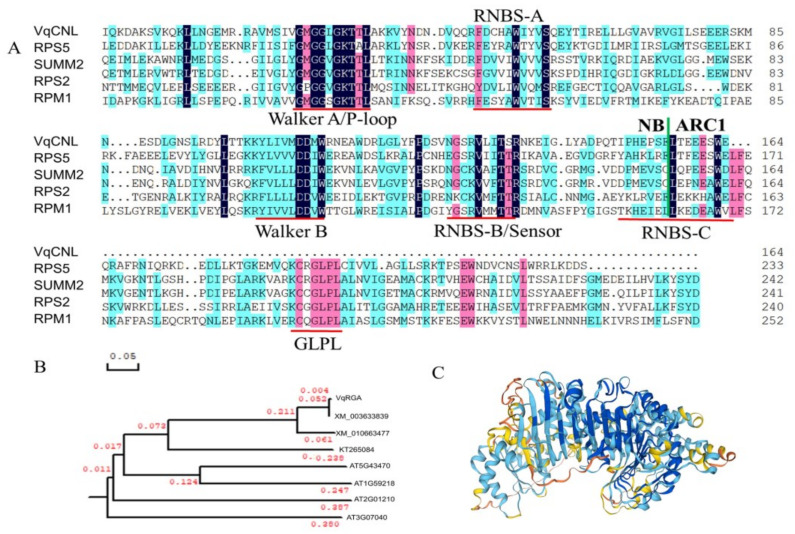
(**A**) Multiple amino acid alignment of the *VqCNL* NB-ARC domain with other related proteins (AT1G12220, AT1G12280, AT3G03600 and AT3G07040). (**B**) indicates phylogenetic tree analysis of *VqCNL* in *V. quinquangularis* ‘Dan-2’ with other related *VqCNL* proteins in plants. The tree was generated by Clustal W method of the MegAlign program. (**C**) indicates structure model of the NB-ARC domain of *VqCNL* in SWWISS-MODEL.

The *VqCNL* gene cDNA fragment, sourced from *V. quinquangularis* ‘Dan-2’, was identified to harbor an open reading frame (ORF) spanning 2697 base pairs. This ORF is responsible for the production of a protein comprising 898 amino acids, with a calculated molecular weight of approximately 102.8 KDa and an isoelectric point (pI) of 7.06. Upon conducting a BLAST analysis, it was established that the *VqCNL* gene is situated on chromosome 15. The structural analysis further predicted the presence of an Rx-CC_like domain within the amino acid sequence range of 11 to 123, an NB-ARC domain extending from residues 154 to 421 and a Leucine-Rich Repeat (LRR) domain spanning from residues 513 to 830 (Figure 1). Additionally, the sequence is characterized by the presence of several conserved motifs, including the P-loop, GFPL, NBS-A, NBS-B, NBS-C, NBS-D and Walker-B motifs, embedded within the predicted protein structure (Figure 2). These findings suggest a complex and potentially significant functional role for *VqCNL* in the molecular mechanisms of the plant, as evidenced by its diverse domain architecture and conserved motifs.

### 2.2. VqCNL Expression Following Downy Mildew Inoculation and SA Treatment

**Figure 3:**
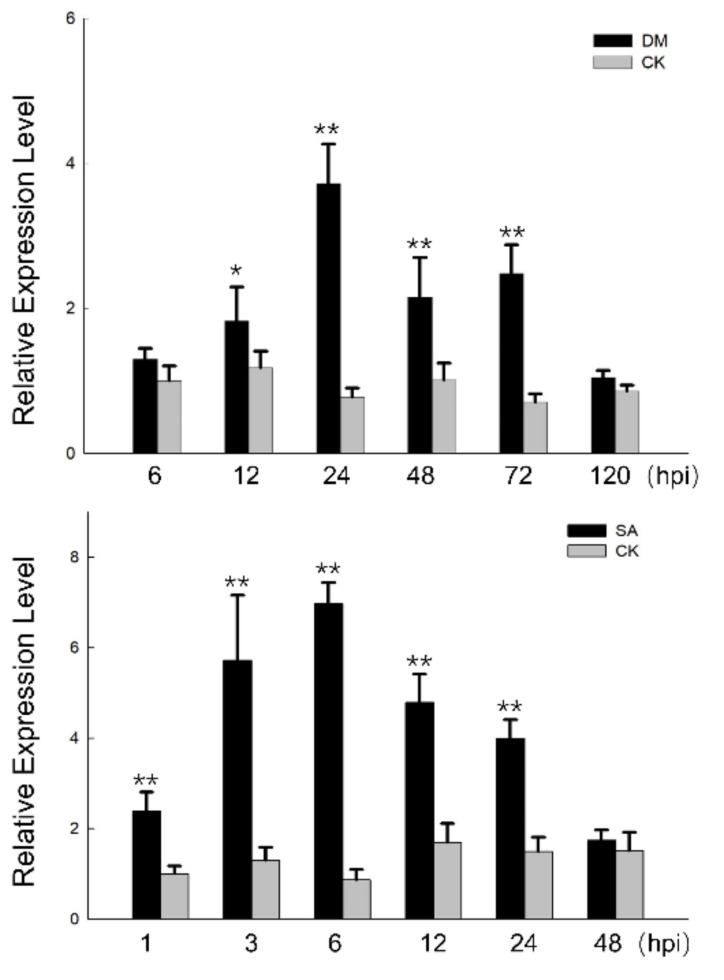
Expression patterns of *VqCNL* in *V. quinquangularis* ‘Dan-2’ following *P. viticola* and SA treatments. Asterisks indicate a statistically significant difference (Student’s *t* test, * *p* < 0.05 and ** *p* < 0.01).

To confirm whether the CC-NBS-LRR gene is associated with DM resistance or not, Chinese wild *V. quinquangularis* ‘Dan-2’ was challenged with *P. viticola*. The expression levels of *VqCNL* experienced a significant increase following inoculation with *P. viticola*, a finding that was robustly confirmed in this study. Moreover, our investigation revealed that the transcription levels of *VqCNL* were markedly elevated after exposure to downy mildew (DM) at four distinct time points (12, 24, 48 and 72 h post-inoculation). Notably, the expression of *VqCNL* reached its zenith at 24 h post-inoculation before demonstrating a gradual decline in the subsequent measurements. We also conducted the experiment to detect whether *VqCNL* was responsive to treatment with SA hormone. The gene expression analysis of *VqCNL* revealed a significant upsurge in its activity following treatment with salicylic acid (SA), peaking at 6 h after the treatment. This heightened expression level began to wane by 48 h post-treatment (Figure 3). This pattern underscores the responsive nature of the gene to pathogenic attack and highlights its potential role in the plant immune response mechanism.

### 2.3. Continuous Expression of VqCNL in Arabidopsis Leads to Increased Resistance against Downy Mildew

**Figure 4:**
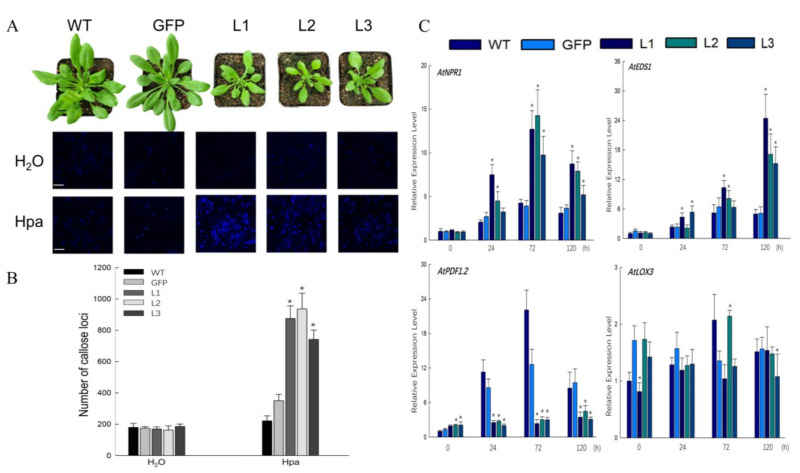
Overexpression of *VqCNL* in *Arabidopsis* enhances resistance to *Hpa*. (**A**) Transgenic *Arabidopsis* plants showed a dwarfed phenotype. Histochemical staining was performed to detect cell death with Aniline blue. (**B**) Biological statistics on the number of callose loci were assessed. Three independent experiments were conducted. (**C**) Expression profiles of defense-related genes *NPR1*, *EDS1*, *PDF1.2* and *LOX3* at different time points after inoculating with *Hpa* in transgenic plants. Asterisks denote a statistically significant difference (Student’s *t*-test, * *p* < 0.05) between the treated plants and their wildtype counterparts. The black scale bars = 10 mm. The white scale bars = 0.5 mm.

**Figure 5:**
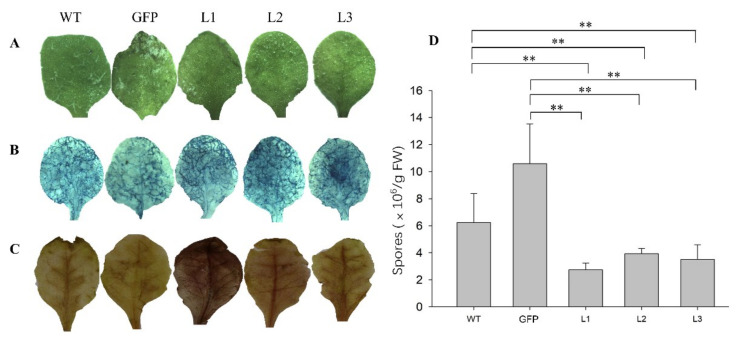
The response of *VqCNL* transgenic *Arabidopsis* lines to *Hpa*. (**A**) Wildtype and transgenic lines (GFP, L1, L2 and L3) 7 days post-inoculation (dpi) with *Hpa*. (**B**,**C**) Histochemical staining was performed to detect cell death, H_2_O_2_ accumulation and callose accumulation at 5 dpi with (**B**) trypan blue and (**C**) diaminobenzidine (DAB) staining, respectively. (**D**) Biological statistics regarding the spore count per gram were evaluated at 5 dpi. Three independent experiments were conducted. Asterisks indicate a statistically significant difference (Student’s *t* test, ** *p* < 0.01).

To delve deeper into the defensive functions of *VqCNL*, its entire coding sequence was fused with the constitutive 35S promoter. The T3 progeny exhibited a dwarfed stature along with the small phenotype (Figure 4A). As a result, three T3 homozygous transgenic lines (L1, L2 and L3) with a normal phenotype and the strongest resistance response to *Hpa* were chosen to use for further study. Compared to wildtype (WT) *Arabidopsis*, three chosen transgenic lines demonstrated a slight disease symptom after *Hpa* infection (Figure 5A). To determine if the enhanced resistance stemmed from the suppression of spore proliferation, we employed a colony counting method to evaluate the growth of *Hpa* on leaves for study. The results, as depicted in Figure 5D, revealed a significant decrease in the number of spores in the transgenic samples (L1, L2 and L3) in comparison to wildtype (WT) plants after *Hpa* infection.

To examine the impact of heterologous overexpression of *VqCNL* on cell mortality, trypan blue staining was applied to infected leaves. The findings revealed extensive areas of cell death in the transgenic leaves, in contrast to the minimal cell death observed in the wildtype plants (Figure 5B). Furthermore, quantitative detection of H_2_O_2_ demonstrated that levels of H_2_O_2_ in three T3 homozygous transgenic lines were higher than that of wildtype plants following challenge with the related pathogen (Figure 4). To detect the deposition of callose after inoculation with oomycete pathogens, infected leaves were chosen and stained with aniline blue. The results showed that, compared with wildtype plants, the amount of callus formation observed in transgenic plants showed a significant enhancement. Further quantitative detection indicates that transgenic plants accumulate more calluses than wildtype plants following pathogen infection.

### 2.4. Expression of Defense-Related Genes

Salicylic acid (SA)-dependent defense mechanisms are crucially involved in enhancing plant resilience against *Hpa* infections, underlining the pivotal role these pathways play in the plant’s innate immune response. As the key genes of the SA signaling pathway, the expression of *AtNPR1* and *AtEDS1* was tested at different time points after inoculation. Both genes exhibited a significantly higher expression level in transgenic lines than that of the wildtype plants before pathogen inoculation. In the transgenic lines, the expression levels of *AtNPR1* and *AtEDS1* peaked at 96 hpi. However, the wildtype displayed different trends and had an observably lower expression level than transgenic lines. Moreover, the expression levels of the nonexpresser of pathogenesis-related protein 1 (NPR1) were assessed using quantitative real-time PCR (qRT-PCR) from 0 to 120 h post-inoculation (hpi) with *Hpa*, to investigate the link between plant resistance enhancement against this pathogen and the activation of such a defense-associated gene. The results demonstrated that NPR1 expression was significantly increased (two-fold) over that of wildtype plants at 72 hpi, but showed a reduction at the time points that followed (Figure 4C).

### 2.5. Constitutive Expression of VqCNL in Arabidopsis Enhances Resistance to Pst DC3000

**Figure 6:**
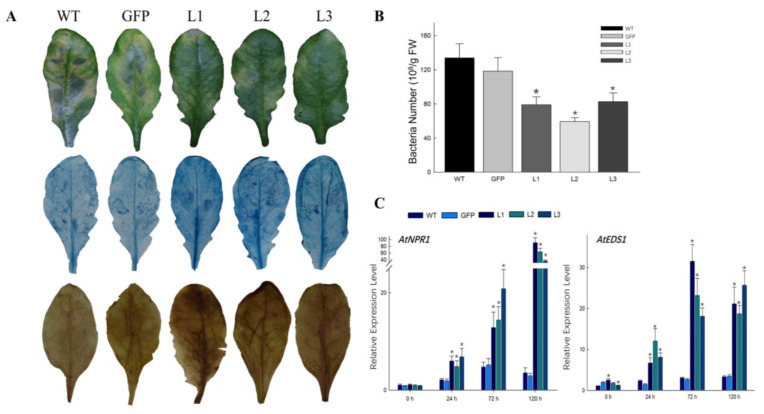
Overexpression of *VqCNL* in *Arabidopsis* improves resistance to *Pst* DC3000. (**A**) The phenotype of transgenic plants following *Pst* DC3000 treatment 5 dpi. (**B**) trypan blue staining was conducted for the cell death detection at 4 dpi. (**C**) Biological statistics of the bacterial number per gram was carried out 2 dpi. The data display average values ± standard deviation (SD) derived from three separate experiments. Asterisks indicate a statistically significant difference (Student’s *t* test, * *p* < 0.05).

A conserved domain, PLN03210, was found within the *VqCNL* amino acid sequence, which is associated with resistance to *P. syringae* [31]. Under this circumstance, we therefore speculated that *VqCNL* might have an effect on improving resistance to bacterial infection of plants. To verify this association, three chosen transgenic lines, alongside wildtype plants, were exposed to *Pst* DC3000, with an evaluation of disease symptoms conducted post-inoculation. Severe symptoms with yellowing leaves and spreading macerations were detected 5 dpi of wildtype plants. However, in the three transgenic lines examined, symptoms observed at 5 days post-inoculation (dpi) were less severe compared to those in the wildtype. To assess the impact of heterologous *VqCNL* expression on cell death, infected leaves underwent staining with trypan blue. The results showed that large clusters of dead cells came into existence in transgenic leaves and few dead cells could be seen in wildtype plants (Figure 6A). As a common experimental method, the colony counting method was employed to evaluate the proliferation of *Pst* DC3000 in infected leaves, aiming to ascertain if the observed increase in resistance stemmed from the suppression of bacterial growth.

As displayed in Figure 6B, the bacterial count was lower in the transgenic lines (L1, L2 and L3) relative to wildtype plants post-infection. The expression levels of the important resistance gene *NPR1* were monitored at various intervals following *Pst* DC3000 inoculation to investigate the signaling pathways contributing to the resistance in transgenic *A. thaliana*. At the same time, this upsurge in *PR1* expression was significantly pronounced, showing a 2–3-fold increase when compared to the expression levels in wildtype plants, reaching a peak at 72h and then decreasing gradually (Figure 6C).

### 2.6. Transient Expression of VqCNL Enhances Tobacco Resistance to Phytophthora capsici

**Figure 7:**
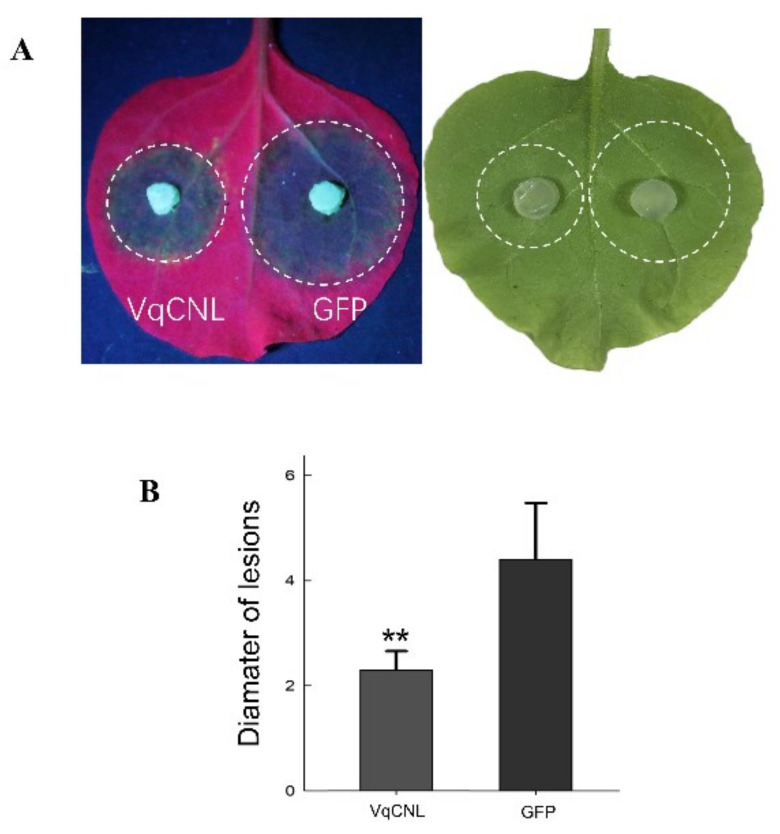
*VqCNL* enhanced resistance to *P. capsi*. (**A**) indicates images of typical symptoms of *VqCNL* and *GFP* which were introduced into *N. benthamiana* through transient expression. Leaves were collected at 48 h after inoculation with *P. capsica*. (**B**) indicates quantification of lesion size in (**A**). The error bars denote standard errors based on three biological replicates. Asterisks signify significant deviations from GFP (** *p* < 0.01, *t* test).

In order to investigate the role of *VqCNL* in plant disease resistance, we used an *Agrobacterium*-mediated transient transformation method to overexpress *VqCNL* gene in tobacco leaves aged 4–6 weeks. The recombinant PHB-*eGFP* vector was used as the control group and inoculated with *P. capsici* two days after injection to detect its disease resistance. Under UV light irradiation, the inoculated detached leaves showed obvious disease spots. *P. capsici* grew slower in transgenic tobacco leaves and the average disease spot diameter was significantly smaller than that of *eGFP* transgenic tobacco leaves (Figure 7). These results indicate that overexpression of the *VqCNL* gene can improve plant resistance to egg-borne pathogens.

## 3. Discussion

Based on our previous report of wild Chinese *V. quinquangularis* ‘Dan-2’ analysis, our research pinpointed a gene believed to be responsible for encoding a CC-NB-ARC-LRR domain, showing a marked increase in activity post-inoculation. In the current research, we extracted the ORF sequence of the gene, now referred to as *VqCNL*, and achieved its continuous expression in *Arabidopsis*. The responses of these transgenic lines to oomycete and bacterial infections were assessed to explain their involvement in response to pathogen attack.

Regulation of gene expression encompasses both transcriptional and post-transcriptional mechanisms. Notably, a significant elevation in the expression levels of various *NB-ARC-LRR* genes has been documented in plants that are infected with pathogens [32,33,34,35,36]. These genes also have been found to be associated with the monitoring of pathogenic microorganisms [37]. In our research, the *VqCNL* gene was tested to be expressed at extremely high levels in Chinese wild *V. quinquangularis* ‘Dan-2’ leaves following challenge with the causal agent of *P. viticola*. Besides, we also found that the expression of the *VqCNL* gene was upregulated in grape leaves treated with exogenous SA. Our findings, following the application of this phytohormone, show a high level of congruence with previous studies conducted on *Arabidopsis* [37,38]. Considering SA’s pivotal role in initiating local defense actions and systemic resistance, our results reveal that *VqCNL* plays a role in bolstering plant disease resistance, operating through the SA signaling pathways. Plenty of research has proved that heterologous overexpression of *R* genes can give rise to plant development retardation and cause related defense activations such as unprompted cell death and a dwarfed phenotype. Scientists speculated that the continuous expression of *R* genes in transgenic plants may cause the over-activation of the ETI reaction [37,39,40,41]. In line with previous reports, our observations revealed that some of our independently developed transgenic T3 Arabidopsis lines exhibited characteristics such as stunted growth, diminutive yellow leaves, and mortality at the seedling stage. These outcomes imply that the expression of *VqCNL*, driven by the constitutive 35S promoter in these transgenic lines, may trigger overly active defense responses and toxic phenotypes in the plants, warranting further investigation into the underlying mechanism.

A number of results indicate that overexpression of *R* genes causes constitutive SA accumulation, upregulation of *PR* gene expression and an active defense response to enhance disease resistance of plants [42,43,44,45]. In our research, heterogeneous expression of *VqCNL* in *Arabidopsis* improved the disease resistance of transgenic plants to *Hpa*. Compared to wildtype plants, the transcript level of *NPR1* observed a two-fold increase in expression at 72 hpi in genetically modified plants, and these levels remained higher over the time course. These results make clear that heterogeneous expression of *VqCNL* in *Arabidopsis* activates defense responses after pathogen inoculation; for example, the overexpression in tomato of *Prf* and *Pto* genes confirmed this effect, as well as *Cf-9* gene overexpression in tobacco [43,46,47]. We also found that *VqCNL* transgenic *Arabidopsis* lines showed broad-spectrum enhanced disease resistance, compared with wildtype plants, to improve the resistance to both the oomycete species *Hpa* and the bacterial *Pst* DC3000. As has been observed previously, the transcriptional level of *AtRPP8* increased rapidly after SA hormone spraying or *Hpa* infection in response to different stresses [38]. Similarly, previous studies showed that the overexpression of *VaRGA1* was first rapidly increased and then decreased slowly after inoculation with *Hpa*, and enhanced disease resistance compared to in wildtype plants [33,34].

Reactive oxygen species (ROS) mainly exist in the form of hydrogen peroxide (H_2_O_2_) and superoxide anions (O^2−^) which play an important role in plant resistance to various pathogens [48,49]. In our results, *Arabidopsis* lines genetically modified with *VqCNL* exhibited a quicker and more substantial accumulation than that of wildtype plants following inoculation with *Hpa* and *Pst* DC3000. It is assumed that this kind of sharp ROS accumulation may directly cause toxicity to the pathogen and lead to increased HR, leading to the death of host cells and prevent the transmission of pathogens [9,50,51]. As a matter of fact, high concentrations of ROS can cause oxidative stress and lead to cell death in plants. In addition, the overexpression of multiple *R* genes in plants has also been found to cause HR-like cell death [52,53]. In this study, we found that the expression of *VqCNL* gene in *Arabidopsis* increased HR-like cell death after *Pst* DC3000 inoculation, indicating that the oxidative burst by infection may activate HR-like cell death and enhance the plant’s immune resistance to pathogens; while it is still possible that the oxidative burst detected in constitutive overexpression lines of *Arabidopsis* may activate additional changes to promote plants’ immunity, including the activation of genes expression related to downstream defense.

Along with ROS, the accumulation of callose is widely found in higher plants and plays an important regulatory role in their life activities. Plants respond to multiple biotic and abiotic environmental stresses through the synthesis and degradation of callose, which works as a physical barrier of plants’ immunity [54,55,56]. The previous research demonstrated that overexpression of the *Pto* gene in tomato increased callose deposition and significantly restricted the growth of pathogenic bacteria [43]. Similarly, as a resistance plant to fungus, the *pmr4* Arabidopsis mutants have been proved to resist penetration of pathogens by the accumulation of callose [57,58]. The results showed that a large accumulation of callose was produced in transgenic *Arabidopsis*, significantly more than in wildtype. As a matter of fact, callose biosynthesis has been shown to be caused by the production of ROS [55]. Since the ROS accumulation level was found to be higher in the *Arabidopsis* transgenic lines than that of wildtype plants, it leads to the speculation that this could contribute to the increased callose deposition in these lines. Nevertheless, further research is still needed to find determinants responsible for callose production in *VqCNL* transgenic lines in response to pathogen invasion.

The screening and identification of functional proteins and finding protein interactions can provide key scientific knowledge for further revealing the molecular mechanism of disease resistance proteins in regulating plant immunity. However, previous studies demonstrated that proteins containing the special domains in grape are highly conserved and act as a key factor in post-transcription regulation of genes and stress response regulation of plants [55,59]. For example, in *Arabidopsis*, a *bHLH84* gene which contains the structural domain of Helix–Loop–Helix changes the disease resistance of plants by directly interacting with the disease-resistant gene *AtRPS4* [41]. In addition, *AtbHLH33* (*SPCH*) plays a key role by regulating the brassinolide pathway during the stomatal differentiation stage [60,61]. In future work, the method of *VqCNL* transmitting disease resistance signals and affecting plant defense response by regulating the expression of related genes in the signaling pathway still needs further study.

## 4. Materials and Methods

### 4.1. Plant Materials and Processing Methods

Chinese wild *V. quinquangularis* ‘Dan-2’, as the experimental materials, were obtained from the greenhouse with appropriate humidity and temperature. The grape plants were inoculated at the pre-bloom growth stage, which is a critical period for grape development and also a stage highly susceptible to pathogen infection. Grape leaves were gathered from the greenhouse and quickly frozen with liquid nitrogen. *Arabidopsis* wildtype seedlings were planted at 20 °C temperatures, with 50% relative humidity, and lighting condition was suitable by providing cool white, fluorescent bulbs.

The pathogen selected for this study was *Plasmopara viticola*, the causative agent of downy mildew in grapes. Pathogen cultures were grown in a controlled environment to ensure virulence. Plants were inoculated under sterile conditions to prevent contamination. A spore suspension of *P. viticola* was prepared at a concentration of 1 × 10^5^ spores/mL. For the inoculated group, each plant was gently sprayed with the spore suspension until runoff. Control plants were sprayed with sterile water. Starting from six hours post-inoculation, samples were collected at 12, 24, 48, 72 and 120 h for detailed analysis. A total of 20 plants were used, divided equally into control and experimental groups.

### 4.2. RNA Extraction and First-Strand cDNA Synthesis

RNA kit production from Omega company was used to extract grape and *Arabidopsis* total RNA as previously described [62]. Grape leaves’ RNA was extracted from sprayed leaves following inoculation with *P. viticola*. Treated by sterile distilled water, leaves as controls were obtained at the same time points. Using the PrimerScript™II 1st Strand cDNA Synthesis kit, first strand cDNA was produced from 500 ng of total RNA (Transgen Biotech, Beijing, China).

### 4.3. Cloning of the VqCNL Gene and Sequence Analysis

The cDNA fragment of *VqCNL* was successfully amplified with the aid of gene-specific primers (refer to Table S1) and Taq DNA polymerase supplied by Qingke Bio. Inc., Shanghai, China. Following amplification, the PCR product underwent cloning into the pLB vector provided by Tiangen Bio Inc., Beijing, China, and its sequence was verified through sequencing conducted by SunnyBio, Shanghai, China, to ensure its correct identity. Analysis of the *VqCNL* nucleotide sequence was performed using the BLASTX tools available on the NCBI website (http://www.ncbi.nlm.nih.gov/BLAST (accessed on 9 September 2023)), while predictions about its chromosomal location within the grape genome were made using the Genoscope Genome Browser (http://www.genoscope.cns.fr/blat-server/cgi-bin/vitis/webBlat (accessed on 9 September 2023)). Further, the protein’s amino acid sequence and its conserved domains were identified using the SMART website (http://smart.embl-heidelberg.de/smart/changemode.pl (accessed on 9 September 2023)). This included deducing the presence of CC, NB-ARC, and LRR domains, which were then compared and aligned with sequences of closely related proteins. Protein structure of *VqCNL* was predicted by SWWISS-MODEL website (https://swissmodel.expasy.org/ (accessed on 9 September 2023)) [63].

### 4.4. Quantitative Real-Time PCR to Test the VqCNL Expression Profile

Primers for quantitative real-time PCR reactions in this part can be found in Supplementary Table S1. Bio-Rad IQ5 real-time PCR detection system (Bio-Rad, Hercules, CA, USA) was used for reaction performance. Each reaction contained 20 µL volume of 1.0 µL cDNA template, 0.8 µL primers, SYBR Taq 10 µL and 8.2 µL sterile water. The reactions were carried out using specified thermal conditions: an initial 30 s at 94 °C, then 45 cycles consisting of 5 s at 95 °C, 30 s at 58 °C and 30 s at 60 °C. Additionally, a melting curve analysis was performed through a program of 40 cycles, starting at 95 °C for 15 s and gradually increasing from 60 °C to 95 °C. For reference in the study, the grape *Actin1* (GenBank Accession No. AY680701) and *Arabidopsis thaliana Actin1* (TAIR: AT2G37620) genes were utilized. All reactions were carried out using three biological replicates, as well as three technical replicates.

### 4.5. Construction of the Heterologous Expression Vector and Generation of Transgenic Plants

To construct the 35S::*VqCNL* heterologous expression vector, the *VqCNL* coding sequence was fused into the pHB-*eGFP* vector, downstream of the *CaMV* 35S promoter, using gene-specific primers. Following the verification of the sequence in the resulting recombinant plasmid, it was electroporated into *Agrobacterium tumefaciens GV3101*. Subsequently, this plasmid, now designated as pHB-*VqCNL*-*eGFP*, facilitated the genetic modification of wildtype *Arabidopsis thaliana* (Col-0) in line with established protocols [64]. From this, transgenic lines L1, L2 and L3, which exhibited the most pronounced expression increase upon *Hpa* induction, were selected to breed T3 generation homozygous plants for further studies.

### 4.6. Pathogens Inoculation of Hpa and PstDC3000

The *Arabidopsis* downy mildew was caused by *Hpa* and the inoculation of this experiment was performed following the previously described method [38]. Seeds from both transgenic and wildtype *Arabidopsis thaliana* were uniformly sown in each pot. Approximately 3–4 weeks later, once the plants developed four leaves, a solution for treating *Arabidopsis* downy mildew was uniformly applied across the foliage. Subsequently, any excess liquid on the leaves was carefully wiped off. After that, the plants were put in a greenhouse and cultured at an appropriate condition for 7 days. Five days after inoculation (dpi), assessments of spore counts and histochemical studies were undertaken. Similarly, evaluations of the area of leaf necrosis and the proliferation of spores were statistically analyzed at 5 dpi.

*Pst* DC3000 was cultured on Luria Bertani (LB) medium which included 50 μg/mL Rif until reached an OD600 of 0.4. After that, bacteria were collected by centrifugation and resuspended in suspension liquid at room temperature. *Arabidopsis* plants were immersed in a cell solution with 0.05% Silwet L-77 for a duration of 15 min, after which they were transferred to a plant incubator set to optimal humidity levels, awaiting the emergence of disease symptoms [65]. Leaves manifesting infection were harvested at various intervals (0, 24, 48, 72 and 96 h post-inoculation) for subsequent analyses. To assess the bacterial population, leaves from distinct lines were sterilized at 2 days post-inoculation. Leaf segments measuring 0.5 × 0.5 cm^2^ were cut out, blended into the cell mixture, and the resultant solution was methodically diluted using sterile water. A volume of 100 μL of this dilution was spread on culture media, and the dishes were then cultivated on a shaker at 28 °C for 48 h.

### 4.7. Trypan Blue Staining, Peroxide Assay and Callose Accumulation

Six weeks *Arabidopsis* rosette leaves were harvested at 12 hpi from four tested lines (wildtype and L1, L2 and L3). Trypan blue solution which included 30 mg trypan blue, 20 mL ethanol, 10 mL lactic acid, 10 mL phenol and 10 mL water was prepared in advance. Leaves were first submerged in boiling water for two minutes and then allowed to sit at room temperature for thirty minutes to absorb the stain. After staining, the leaves were clarified at 25 °C throughout the night and subsequently conserved in a solution of 70% glycerol. A compound microscope was used to examine the stained leaves [66,67,68]. Three independent experiments were performed.

The assessment of H_2_O_2_ production in *Arabidopsis* was conducted using diaminobenzidine (DAB) staining. Rosette leaves from both wildtype and the L1, L2, and L3 transgenic lines were gathered at 24 h post-inoculation (hpi) and subjected to staining through a 10 min vacuum infiltration in a 2 mM DAB solution, prepared in 20 mM phosphate buffer at pH 6.0. This was followed by a 1.5 h incubation at room temperature. Afterwards, the leaves were placed in a 95% ethyl alcohol solution and incubated in a water bath at 80 °C for 20 min to clear the chlorophyll. Samples preserved in distilled water were subsequently photographed for analysis [69].

To evaluate callose deposition in transgenic Arabidopsis strains, six rosette leaves each from the wildtype and transgenic lines L1, L2, and L3 were harvested at 3 days post-inoculation (dpi) and soaked overnight in a mixture of acetic acid and ethanol in a 1:3 ratio. After soaking, the leaves were thoroughly rinsed multiple times with 150 mM K_2_HPO_4_. Following the rinse, the leaves underwent staining for one hour in a solution of 150 mM K_2_HPO_4_ and 0.01% aniline blue, before being preserved in 50% glycerol [70]. The detection of callose deposits was then performed under a Zeiss fluorescent microscope (Leica, Weztlar, Germany) equipped with the necessary filters for fluorescence observation [71].

### 4.8. Instantaneous Expression Transformation of Tobacco

In the experiment, *Nicotiana benthamiana* seeds were vernalized at 4 °C for one week and planted in the substrate until seedlings emerged, and then transferred to an incubator for cultivation for 4–6 weeks. After the tobacco leaves expanded and grew, they were prepared for instantaneous transformation analysis. The oat culture medium was used to inoculate the *Phytophthora capsica* for 5–7 days at room temperature. A punch with a diameter of 7 mm was used to punch the medium. The mycelial surface of the bacterial disk was placed tightly against the back of the tobacco leaf. After sealing the culture dish and incubating it in dark conditions for 2–3 days, the phenotype was observed, photos taken and the size of the lesion recorded. Three repeated experiments were conducted and more than 6 tobacco leaves were used in each replicate.

### 4.9. Statistical Analysis

All experimental procedures were independently repeated three times, utilizing three biological replicates for each. The outcomes are displayed as mean values and standard errors, analyzed through the SigmaPlot 10 software. To further validate the statistical significance of the observed differences between experimental groups, paired t-tests were carried out using the SPSS Statistics 17.0 software (IBM China Company, Shenzhen, China).

## 5. Conclusions

At present, there are few reports on *R* genes with broad-spectrum resistance, which cause the restricted development of molecular breeding. While several reports have found that constitutive overexpression of NBS-LRR genes can enhance the SA signaling pathway and improve the expression of defense-related genes to activate plants’ broad-spectrum immune resistance. In conclusion, our results preliminarily demonstrated functions of the *VqCNL* gene to both confirm resistance to both oomycete and bacterial pathogens in plants via upregulation of defense-related genes, and activate the SA signaling regulation pathway. Our research also made some contribution to identifying and characterizing NBS-LRR type genes with broad-spectrum resistance function. Furthermore, detailed mechanisms of eliciting downstream defense responses need to be further investigated. Future studies will focus on the investigation of possible interactions between *P. viticola* effector proteins and the *VqCNL* gene. This will supply useful information on the molecular mechanism with the high level of downy mildew resistance displayed in Chinese wild *V. quinquangularis*.

## Data Availability

Data is contained within the article and Supplementary Materials.

## Supplementary Materials

The following supporting information can be downloaded at: https://www.mdpi.com/article/10.3390/ijms25063221/s1.
